# Supplementary material for: Unravelling reference bias in ancient DNA datasets
Source: Bioinformatics. 2024 Jul 3;40(7):btae436. doi: 10.1093/bioinformatics/btae436 (PMC11254355; doi:10.1093/bioinformatics/btae436)

verysensitive_bowtie_rhino_div9_1end

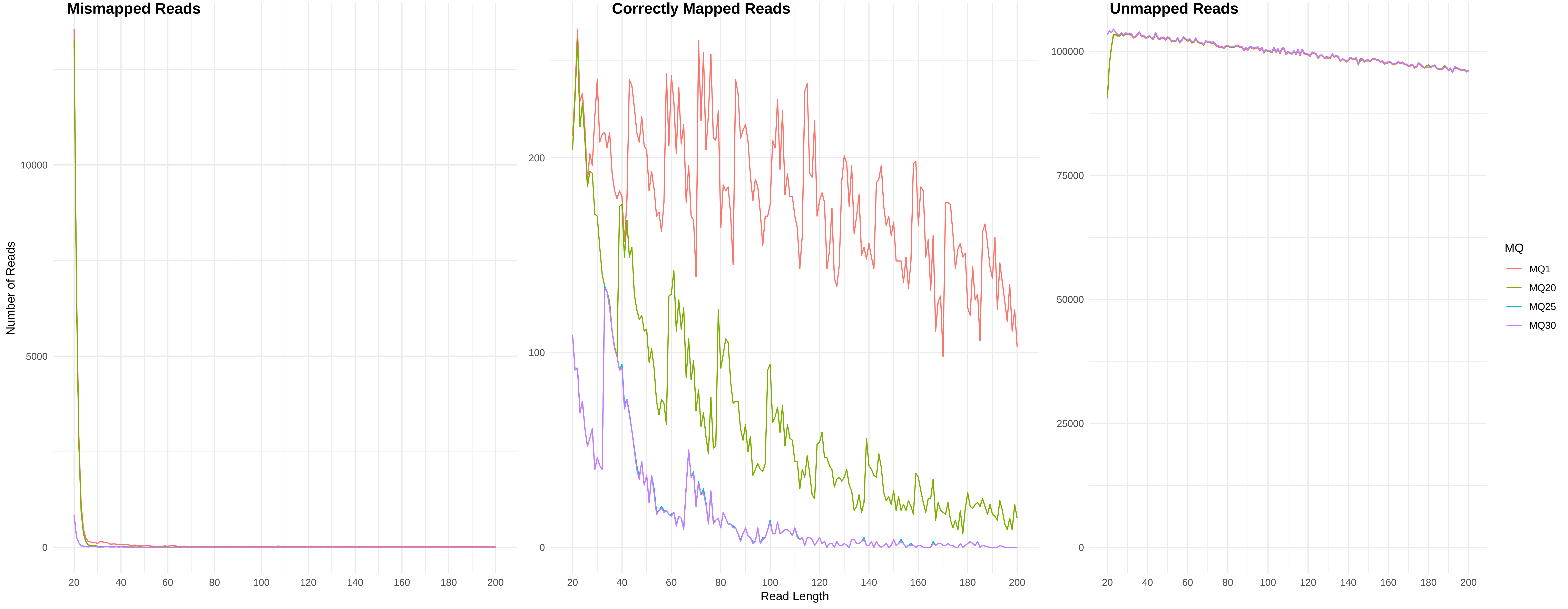

Supplement: btae436_Supplementary_Data [file btae436_supplementary_data.zip › S1-S4_data_plots.zip!/S1-S4_data/mapping_stats_plots.zip!/mapping_stats_plots/verysensitive_bowtie_rhino_div9_1end_mapping_stats_byMQ_plots.pdf]
